# Minimally invasive total knee replacement: techniques and results

**DOI:** 10.1007/s00590-018-2164-4

**Published:** 2018-03-22

**Authors:** Frederic Picard, Angela Deakin, Navin Balasubramanian, Alberto Gregori

**Affiliations:** 1 Golden Jubilee National Hospital, Agamemnon Street, Clydebank, G81 4DY UK; 2 Biomedical Engineering, Strathclyde University, Glasgow, UK; 3 Surgiconcept Ltd, Glasgow, UK; 4 Hairmyres Hospital, Eaglesham East Kilbride, Glasgow, G758RG UK

**Keywords:** Minimally invasive surgery, Less invasive surgery, Total knee replacement, Total knee arthroplasty

## Abstract

In this review, we outlined the definition of minimally invasive surgery (MIS) in total knee replacement (TKR) and described the different surgical approaches reported in the literature. Afterwards, we went through the most recent studies assessing MIS TKR. Next, we searched for potential limitations of MIS knee replacement and tried to answer the following questions: Are there selective criteria and specific patient selection for MIS knee surgery? If there are, then what are they? After all, a discussion and conclusion completed this article. There is certainly room for MIS or at least less invasive surgery for appropriate selected patients. Nonetheless, there are differences between approaches. Mini-medial parapatellar is easy to master, quick to perform and potentially extendable, whereas mini-subvastus and mini-midvastus are trickier and require more caution related to risk of haematoma and vastus medialis oblique (VMO) nerve damage. Current evidence on the safety and efficacy of mini-incision surgery for TKR does not appear fully adequate for the procedure to be used without special arrangements for consent and for audit or continuing research. There is an argument that a sudden jump from standard TKR to MIS TKR, especially without computer assistance such as navigation, patient-specific instrumentation or robotic, may breach a surgeon’s duty of care towards patients because it exposes patients to unnecessary risks. As a final point, more evidence is required on the long-term safety and efficacy of this procedure which will give objective shed light on real benefits of MIS TKR.

## Introduction

In the early 1980s, laparoscopic abdominal surgery undeniably pioneered minimally invasive surgery (MIS) in assessing, then promoting and finally demonstrating favourable benefits such as pain reduction and early patient recovery [1], since many studies have documented reduced length of hospitalization stay [2], early recovery time and reduction in complications in laparoscopic surgery [3].

At the end of the 1990s, following these general surgery concepts, a few orthopaedic surgeons advocated the use of MIS in unicompartmental knee replacement (UKR) [4]. Actually, the concept of MIS knee replacement commenced long after mini-invasive hip surgery [5, 6] when the Judet brothers described a less invasive anterior hip approach using Hueter principles. However, the true concept of MIS arose later at the beginning of this century mainly driven by the two-incision hip approaches [7, 8]. Surfing on the MIS model for hip, several teams [9, 10] disseminated minimally invasive total knee replacement (TKR). This concept also coincided with advanced development in computer-assisted technology [11–14] and with the resurgence of UKR [15, 99].

Early results in mini-incision in total hip replacement (THR) proved reduced post-operative pain and blood loss [16]. Later, authors evaluated MIS TKR and confirmed that post-operative pain was reduced compared to a conventional TKR approach, but also it improved earlier knee motion, lessen blood loss and shorten hospital stay [17]. Pioneers in this field defined the precepts and goals of minimally invasive knee replacement such as reducing the skin incision and mobilizing the muscular skin window while performing surgery [10]. Also, they pointed out the restrain of disruption of the supra-patellar pouch and recommended avoiding quadriceps tendon incision as little as possible either using mini-parapatellar [18] approach or subvastus approach to facilitate early quadriceps strength and straight leg raise [19, 20]. They avoided everting the patella or manipulating the surrounding soft tissue structures by using additional assistants or special retractors for faster knee flexion recovery [21, 22]. This gave the concept of mini- and less invasive TKR without clearly defining the difference between the two. However, both claimed to reduce soft tissue damage, consequently reducing blood loss, pain and improving early patient recovery, mobilization and subsequently enhancing the functional outcome [23].

Conversely to the very encouraging results listed previously, some authors raised some concerns with MIS TKR regarding component malalignment [24], skin damage [25], denervation injury through midvastus or haematoma through subvastus approaches [26]. Even worse complications, such as peroneal nerve damage [27], patella tendon rupture [28] or acute compartment syndrome [29], were attributed to the lack of vision of the lateral compartment in particular.

In this review, we will outline the definition of minimally invasive and less invasive approach in TKR; then, we will describe the different surgical approaches reported in the literature. Afterwards, we will go through the most recent studies assessing MIS TKR and identify the advantages and drawbacks of the described techniques. Next, we will search for potential limitations of MIS knee replacement and try to answer the following questions: Are there selective criteria and specific patient selection for MIS knee surgery? If there are, then what are they? Finally, a discussion and conclusion will complete this article.

## Definition MIS in knee replacement

The first description of TKR approaches originated from knee trauma when obviously the use of extensive approaches was not an issue, it was a necessity. Some of these approaches were used at an early stage of arthroplasty such as the medial parapatellar or subvastus approaches or the lateral approach of Keblish to quote only the most commonly used ones [30–33]. Repicci et al. [4, 34] endorsed UKR and promoted minimally invasive approach in reducing the skin incision to around 10 cm length centred on the affected compartment and in the same time reducing the medial parapatellar approach to the minimum necessary. Later, pioneers in MIS [9, 10, 26] used already known approaches and reduced their invasiveness to fit the definition of minimally invasive or less invasive surgery (LIS). The medial parapatellar became quadriceps sparing approach [9, 28] and mini-medial parapatellar approach [18], the midvastus became mini-midvastus [21, 22, 35, 36], and the subvastus became mini-subvastus [19, 20, 37, 38]. It was quickly evident that standard instrumentation was not adequate for MIS, so new instrumentations including computer-assisted surgery and even new implant designs emerged and were used to facilitate the surgical procedure and launch the new trend of MIS [36, 38]. Concomitantly, new anaesthetic techniques including spinals [39], peripheral blocks [40] and local infiltrations [41] were instituted into standard care and amplified MIS outcomes and benefits. Surgical and anaesthetic techniques, new technology and new instrumentations all acted conjointly in the concept of MIS or less invasive surgery (LIS) [26].

MIS and its aims can be confused. MIS is about “tissue sparing” surgery that lessens the assault on the joint and the patient so giving a better outcome [36]. It aligns with the Enhanced Recovery After Surgery (ERAS) philosophy of doing less damage so that the patient recovers quicker [42]. In some ways, the title MIS is unfortunate as it leads to a focus purely on incision length (easy to measure and report) and the associated improved cosmesis [36]. It is often confused with minimal or mini-incision surgery where the same cuts are made under the skin as in a standard approach, but the skin incision is smaller. This does not fulfil the aims of MIS. In an ideal MIS tissue sparing surgery the tissues are cut, displaced and handled as little as possible during the operation so as to minimize the damage to them and working with tissues the minimum force is used. Less damage caused during the operation should correlate with a quicker recovery and better function [43].

We will now describe in detail each MIS approach.

## MIS approaches description

**Fig. 1:**
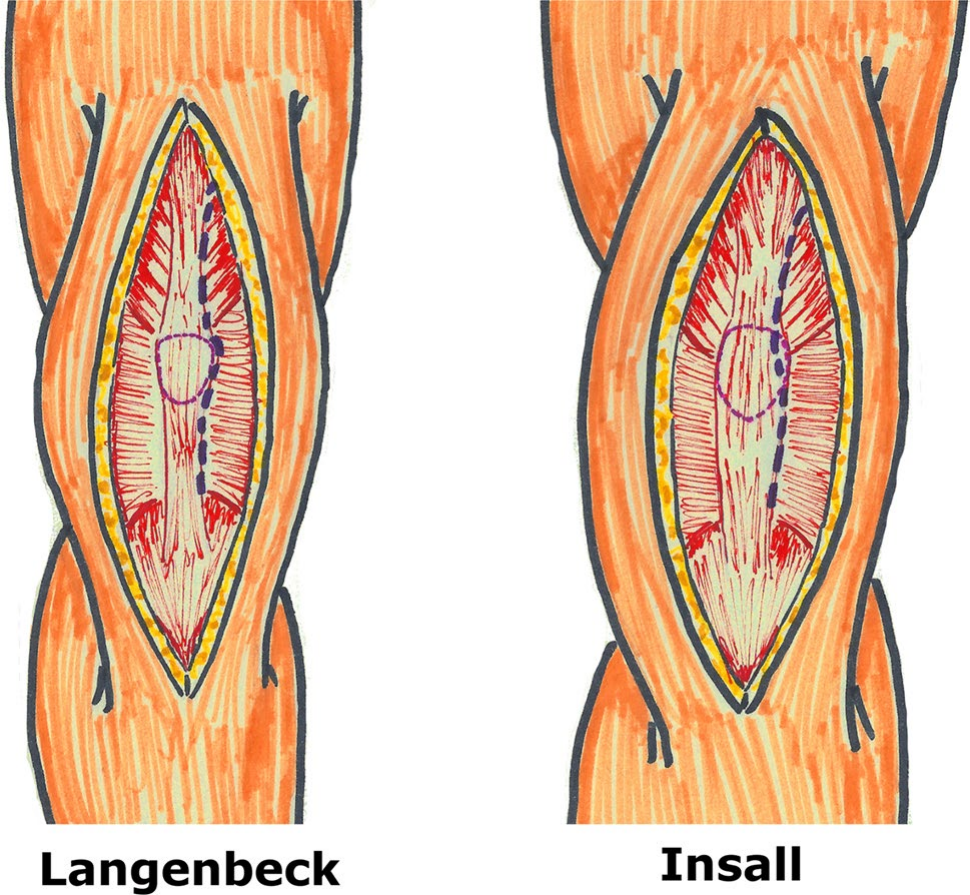
Conventional approaches of Langenbeck (left) and Insall (right)

The more usual anterior knee approaches are the two described by Langenbeck (in 1879) and Insall opening the joint medially along the vastus medialis oblique (VMO), close to the patella and the patellar tendon for the later [44] (Fig. 1).

**Fig. 2:**
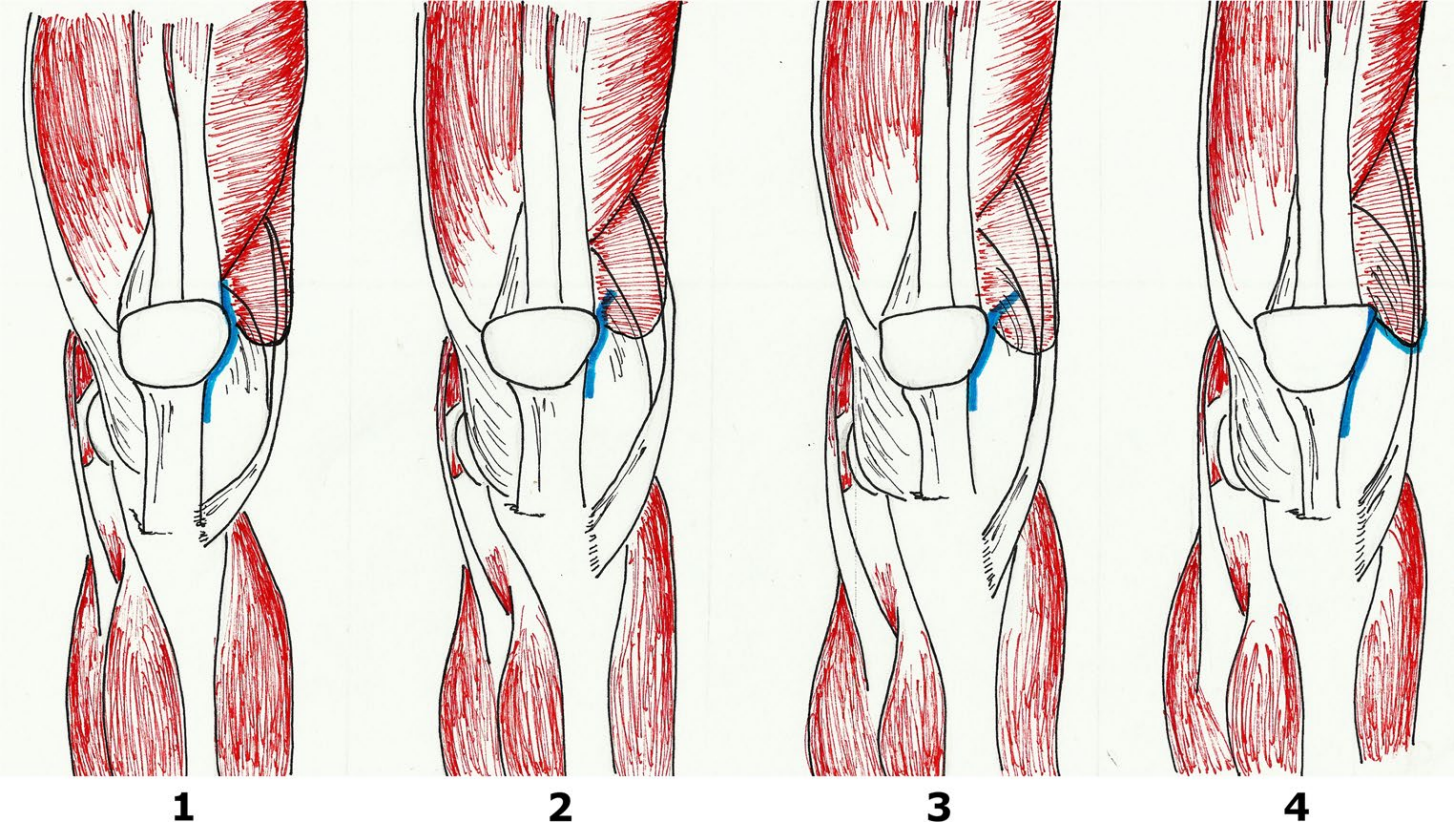
From left to right: (**1**) mini-medial parapatellar approach which is basically a small conventional approach without cutting the quads; (**2**) MIS Quad-sparing approach which is straight above the patella, (**3**) mini-midvastus approach which goes through the vastus medialis approach (VM); (**4**) mini-subvastus approach which goes under the VM

Four mini-invasive approaches are shown in Fig. 2.

The mini-medial parapatellar is a modification of a standard TKR approach. It was first put forward by Tenholder et al. [18] and is based on the idea that approaches are on a continuum from the tradition to the quadriceps sparing. The authors used a standard medial parapatellar approach and arthrotomy but defined the “MIS” version as a limited skin incision, less than 14 cm, limited medial parapatellar arthrotomy and an incision into quadriceps tendon limited to 4 cm [18]. Results are reported to be similar but not identical to MIS. However, it is useful as it allows the surgeon to judge the necessary exposure from minimal to large. It requires less training than other MIS approaches and is easy to extend if lack of exposure becomes a problem during the operation [26]. Further limitation of the incision into the quadriceps tendon (2 cm or less) makes this approach comparable to the quadriceps sparing, giving the same clinical results [45].

The mini-midvastus approach was first reported in 2004 [17, 21, 46] and is the most popular approach for MIS in the knee [26]. It limits the division of vastus medialis to 2 cm, and the patella is subluxed but not everted. The skin incision extends from the superomedial pole of the patella to just below the joint line. This approach is a traditional medial parapatellar capsulotomy extended to the fibres of the vastus medialis [46]. The approach is a modification of the familiar midvastus, does not require major instrument modification and is extensible in difficult cases [26]. However, this could lead to possible denervation of parts of the vastus medialis, vasculature disruption and haematoma formation, but in general these problems are not seen if the division is kept to 2 cm or less [26].

The mini-subvastus approach has no quadriceps incision; rather, the muscle is raised across the anterior aspect of the femur and retracted laterally. The incision is made along the inferior border of vastus medialis and then distally along the medial side of patella [47]. Early papers identified this as a difficult technique with proper patient selection required [48]. This approach is meant to completely avoid damage to the quadriceps mechanism, but it showed similar post-operative outcomes to the mini-midvastus [47] and quadriceps sparing [49]. It is a particularly difficult approach with a large quadriceps muscle mass so not suitable for muscular male patients; it is not currently popular [26].

The quadriceps sparing approach was first reported in 2003 [9]. The idea, as the name suggests, is to avoid damaging the quadriceps tendon and muscle and so they are not divided. It is defined as a skin incision of around 10 cm in length, and the arthrotomy extends from the superior pole of the patella to 2 cm below the tibial joint line on the medial side of the knee. Instruments to enable this were new and unfamiliar, so surgeons required cadaveric training before performing this approach [26]. Early good results were reported but not for all patients or all surgeons—patient selection and skill of surgeon were vitally important for this approach to be used successfully [26]. Some prostheses were modified to fit with this approach, being made smaller. Results improved with time [36, 45].

## Compare conventional surgical technique vs MIS

The initial literature supporting MIS gave earlier recovery, less pain, greater flexion, less blood loss [17, 23, 43, 50]. However, other authors raised concerns about increased complications including malalignment and wound problems [24, 25, 28] and some did not identify significant differences with respect to conventional TKR for blood loss, infection or ultimate wound healing [51].

As MIS TKR has been around for a while, there is enough published evidence for meta-analyses to be carried out. However, results are still not conclusive. In general, MIS approaches appear to give better earlier flexion and/or range of motion [52–58] although not for all MIS approaches [52, 53]. The majority showed earlier straight leg raise [52, 53, 55] but not all [54]. MIS appears to improve immediate post-operative pain scores [52, 54] and early KSS [52, 53, 57] although one analysis found no difference in KSS [54]. Based on the current meta-analyses [52–58] there are conflicting results for blood loss, LoS, wound problems and peri-operative complications between MIS and standard approaches, so it is still not easy to draw conclusions. However, two of the most recent meta-analyses have shown no clinical advantages for the quadriceps sparing approach over the medial parapatellar but an increased risk of malalignment and malposition [59, 60].

Another issue with the success of MIS TKR is the fact that it is only one part of a major change to care over the last 15–20 years [26]. The changes outside the actual surgical procedure have been termed enhanced recovery programmes (ERP) and often include local infiltration analgesia (LIA) and have shown early mobilization, short length of hospitalization stay and good functional outcome results easier recovery, shorter hospital stay, less pain, less blood loss, greater RoM [61–63]. Therefore, it is hard to isolate the effect of MIS and similar results can be achieved without its use [64]. However, MIS TKR may still show better range of knee motion after the third day [65].

## Potential limitations of MIS

Minimally invasive knee surgery implies a restricted visual field with respect to a standard knee approach whatever approach is chosen. The concept of MIS or less invasive surgery is associated with the technique of “movable window” which allows the surgeon to access anatomic articular structures in moving the knee until the surgeon’s axial vision is centred on the interested area through the open soft tissue of the knee [21]. It is straightforward to understand that medial knee anatomic structures are easily accessible with any minimally invasive medial approaches.

**Fig. 3:**
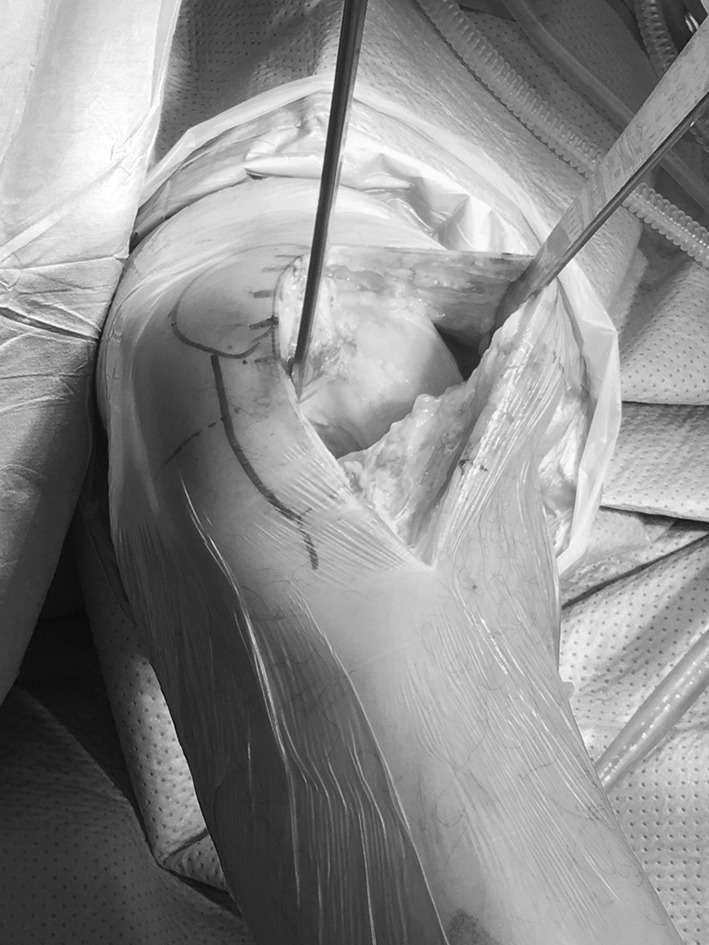
Field of view of the mini-parapatellar approach

Indeed, the mini-subvastus approach offers a nice view on the medial aspect of the knee [17] as well as mini-midvastus and even mini-parapatellar approaches extended to 2 [45] or 4 centimetres [18] (Fig. 3). On the other hand, it is easy to recognize that the lateral side of the knee is trickier to access with any of the medial approaches even with mini-instrumentations and good lighting.

The fad pad that some are preserving to increase post-operative flexion [67] and avoid post-operative patella baja may reduce visibility on the lateral femoral condyle and tibial plateau. However, new instrumentations have been designed to offset the lack of access [36]. Also, lighting apparatus either affixed on the surgeon’s helmet or directly on retractors appeared with advanced MIS techniques. In order to increase the vision on the lateral aspect of the joint, some surgeons advocated performing the first step of patella resurfacing, i.e. osteotomy, which then opens up the field of view [46, 68]. As mentioned already most of the techniques encourage the surgeon not to evert the patella so to not close the lateral compartment of the knee to the view [26, 69].

However, even with appropriate instrumentations, deep anatomic structures of the knee are very difficult to visualize such as the popliteus tendon and particularly it’s femoral insertion, but also the posterior horn of the lateral meniscus, the lateral synovium tissue and even more difficult to see are the ligament collateral ligament (LCL) and the biceps tendon [27]. Despite the small MIS field of view, surgeons may still see reasonably well in slim and easy knee, but it becomes more challenging with overweight patients, with stiff knee and/or patella baja [70].

In this section, we would like to identify in the literature some of the anatomic structures that may compromise the surgeon’s field of view and ultimately infringe some of the basics principle of TKR.

### Skin issues

The first limiting structure of MIS is the skin. MIS or LIS requires stretching the skin particularly to both extremities of the incision. Studies showed that stretching the skin generated ischaemic areas that may not recovered especially during long period of time [71], such prolonged surgical duration [48]. Consequently, the skin may struggle to recover even after thorough closure and can compromise early healing [51]. Seo et al. [72] reviewed a series of 448 knees MIS TKR and found that minor wound complication rate was 13% with fat pad resection and 3% with infrapatellar fat pad preservation.

Skin length incision is a central objective of MIS or LIS TKR. The position, alignment and stretch yield to the skin incision are essential to understand in the MIS TKR. The Langer’s line on the skin knee is horizontal roughly in the front of the patella and slightly above, whereas they become vertical beyond. “Lines of maximal extensibility run perpendicular to the relaxed skin tension lines or Langer’s line and indicate the direction in which closure can be performed with the least tension”. Thus, there will be less tension at the edge of a wound in the same orientation of relaxed skin tension lines [73, 74]. The small skin incision of MIS usually remains in the horizontal skin tension line. It means that long-term stretches on the elastic fibres may result in the loss of recoil because elastin can fragment and compromise closure and early healing [71]. “This especially happens after 70 years old” which represent a big contingent of knee replacement age group [75]. Some are recommending assessing skin extensibility with the “pinch test” prior to perform the skin incision. MIS principles rely on limited skin incision which may put at risk the surrounding structures and may compromise skin blood flow [74]. Overall these factors may explain some of the wound issues after MIS TKR.

### Muscles and underneath skin tissue issues

Underneath skin tissues (i.e. musculo ligamentous structures) are subject to sturdy tension either by the surgeon, assistant(s) or instrumentations during MIS TKR. Niki et al. [76] noted that the midvastus approach created the greatest rise in myoglobin and creatinine kinase levels. However, it seems that torque measurements [77] and electromyography [EMG] analysis [78] could not find any differences between midvastus and subvastus approaches which is an extra-muscular approach. Callaghan et al. [78] observed that EMG abnormalities in the quadriceps muscle are certainly neuropraxic injury but with no functional consequences. In another study from Kelly et al. [79], they also were not able to identify any difference between medial parapatellar and midvastus splitting approach using again EMG exploration. Therefore, stretch and tension exerted on the muscular tissues whatever approaches are used may have biologic or EMG consequences but apparently no functional aftereffects.

According to Niki et al. [76] again from the perspective of the kinetics of muscle-related enzymes, the degree of muscle damage was actually equivalent between MIS and conventional TKR. Therefore, MIS may actually not be much different from conventional surgery as far as tissue damage is concerned.

### Patella tendon and vascular nerve structure issues

In 2011, a meta-analysis compared complications between MIS and conventional knee replacement and they clearly found increasing number of complications with MIS compared to conventional TKR [27]. Indeed, MIS surgery seemed to demonstrate greater prevalence of skin necrosis and delayed wound healing with more superficial wound and even more deep infections, three times more than conventional TKR. We already went through potential reasons for these eventful skin complications, but more worryingly are other very serious complications such as patella tendon injury, partial or even complete section, twice as frequent in MIS than conventional TKR. It is interesting to note that popliteus tendon injury, deep peroneal nerve palsy and maybe notching are also more recurrent in MIS TKR, which might be related to reduced view of the lateral compartment.

Popliteus tendon damage has been reported in the literature with conventional knee replacements, but probably innocuous to patients [80] and also insignificant on knee laxity in particular [81]. As a result, we could consider this complication as minor, whereas the patella tendon rupture is unquestionably more serious as well as deep peroneal nerve injury which have both severe consequences and sometimes definitive impairment for patients. MIS recommends to not evert the patella to avoid stuffing the joint line, but actually a study showed that out of 66 randomized TKR everting or not the patella did not impact the post-operative patella height but did increase its risk of damage [82].

For Gandhi et al. [27], the MIS TKR appears to cause unnecessary risk for the patient without demonstrating any clinical benefit in the parameters examined. It is true that some studies are alarming and reporting for instance vascular damage which are obviously related to the lack of vision and limited access to the posterior aspect of the knee. Some surgeons advocated to keep the knee in extension or in midflexion to saw the tibia without any posterior retractor protection increasing obviously the risk of posterior vascular damage [68].

Finally, in a study from Kuo et al. [83], they reported a high transfusion rate which was 39.5% (81 patients out of 205) in MIS group and 8.3% (34 patients out of 410) in the control group (*P* < .001). Longer surgical duration amongst other factors may explain the difference.

It seems that knee soft tissues are at risk when performing MIS TKR.

### Cementation issues

The lack of sight can also compromise the cementation if used. Again, Ganghi et al. [27] found in their review an increase in gross component malpositions, subsidences and more non-progressive radiolucent lines in MIS than in conventional TKR. Ang et al. [84] found similar results even though they could not identify more abnormal cement mantle discrepancy on the lateral tibia, which is the visionless area in most frequently used medial approaches. However, maybe the sample size was too small to identify this problem.

### Instrumentation and implant

**Fig. 4:**
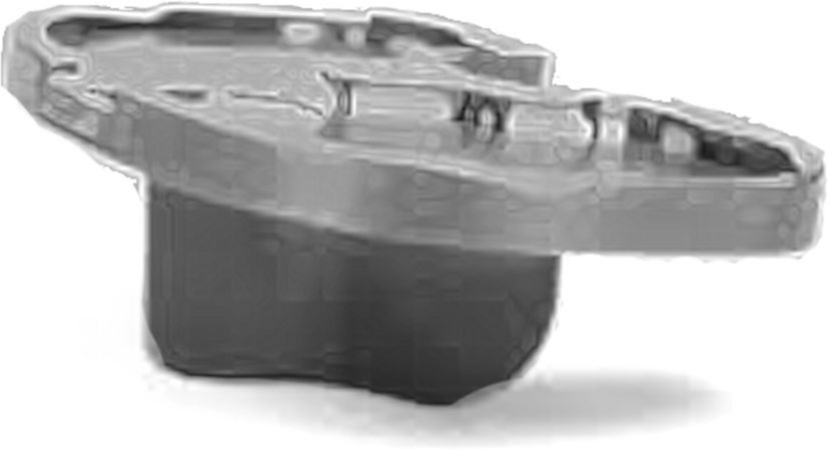
Example of tibial plateau with small keel fitting into minimally invasive surgical (MIS) approach

In order to counterbalance some of the difficulties of MIS TKR, authors have recommended the use of specific instrumentation dedicated to MIS and have even advocated working with more than one assistant [21, 26]. Besides new retractors, companies have redesigned conventional jigs such as the so-called sided cutting tools. Conceptually appealing, these instruments are less cumbersome but still require special assessment because surgeons have to adapt to a new way to place their cutting jigs and performed bone cuts. Conventional instrumentation usually divides the femoro-tibial bony cuts into coronal and sagittal, whereas sided jigs combine both plans in one, which requires further training. There is no proper independent assessment of these jigs. That is why navigation systems and/or patient-specific instrumentation (PSI) gained popularity within the new paradigm [85–88]. In addition to new instrumentation, new implant designs such as short uncemented tibial keels were launched promptly onto the market to respond to MIS constraints related to the small working field of view (Fig. 4). Precoated stems and modular implants were also tried.

Despite noticeable improvements with these instruments, there are still many unsolved questions related not only to technique and material but also to safety, efficacy, cost-effectiveness and clinical advantages of these new tools and new implants [89]. Durability is finally of paramount importance in joint replacement surgery, and therefore, studies must address short-term and long-term results especially with new implants combined to new approaches. Most recent studies between 2 and 4 years and even after 6-year follow-up did not demonstrate negative long-term functional results of MIS TKR with respect to conventional TKR [90, 91]. The two quoted studies included small number of patients with 48 and 66 patients, respectively.

Lastly, Bendich et al. [66] compared two minimally invasive approaches, quadriceps sparing (QS) and mini-subvastus (MS), and of all radiologic measurements, they found that coronal plane tibial component position was outside the desired range in 17.5% of patients in the MS group and 16.4% of patients in the QS group which are both important number of outliers.

Computer-assisted navigation and patient-specific instrumentations are advocated in most of recent reported series of MIS TKR in order to ensure alignment during surgery [106–109]. However, this does not prevent improper cementation to the lack of view, which encouraged companies to develop new uncemented knee designs.

## Are there selective criteria and specific patient selection for MIS knee surgery?

The initial papers about MIS in UKR gave clear patient selection criteria [34]. Some authors have deliberately excluded some patients from MIS TKR. In 2006, Lonner et al. [43] defined good and bad candidates for MIS TKR. Suited patients were non-obese, non-muscular (mainly female), with varus deformity < 10°, valgus deformity < 15°, range of motion (ROM > 90°) and inadequate patients were those with stiff knees (< 50° ROM), fragile skin, marked obesity, patella baja, large femoral or tibial size, muscular patients, severe osteopenia and rheumatoid arthritis.

In 2017, Khakha et al. [92] confirmed some of these exclusion criteria and adding more such as, fixed flexion deformity > 5°, knee flexion < 90°, valgus deformity > 10°, gross bone loss, patients undergoing revision surgery previous osteosynthesis involving the knee, peripheral vascular disease, diabetes mellitus, long-term corticosteroids and neurological deficit, which ultimately reduced significantly the number of suitable patients for MIS TKR. Finally, other surgeons define the ideal patient as young, thin, healthy and motivated and declined patients who are overweight, with marked bone or joint deformity, muscular and again patients requiring larger sized implants [26, 38].

Nothing in these papers have clearly mentioned a BMI threshold for MIS TKR. However, it is relevant to notice that many published articles are reporting low BMI (< 35). However, Klingenstein et al. [93] evaluated the impact of morbid obesity (BMI > 40) on 597 patients out of 4173 and demonstrated a significant increased length of stay (LOS) compared to MIS TKR performed on lower BMI.

Finally, Amanatullah et al. [94] indicated that due to learning curve and investment in instrumentation, MIS TKR is best suited to specifically trained high-volume surgeons.

## Discussions

Aesop^1^ a famous story teller wrote: “The smaller the mind the greater the conceit”. Would this apply to minimally invasive TKR? “The smaller the approach the greater the conceit?” Certainly, pride may have a bit to do with trying in an already sometimes challenging surgery to make it more complicated by voluntarily reducing the field of view. On the other hand, it is of its time to minimize the invasiveness of surgery. In 1994, Wickham reported on future developments in a paper entitled “minimally invasive surgery” and quoted pioneers in the field of abdominal surgery writing that the general aim of minimally invasive treatment was “to minimize the trauma of interventional process but still achieve a satisfactory therapeutic result” [95].

Mark Twain^2^ wrote “Many a small thing has been made large by the right kind of advertising”. and this is truly what happened with MIS THR and TKR. Callaghan et al. [96] reviewed 92 websites of knee society members and found that 22.7% made indirect reference to MIS TKR, while only 10.9% made a direct reference. They referred to faster recovery on 90% of indirect sites and 50% of direct sites, whereas specific risks were discussed on only 35%. Some companies drove changes and MIS TKR followed the successful introduction of MIS THR. The concept of MIS was obviously very appealing to patients for cosmesis reason and ultimately to surgeons. In 2016, Dalton et al. [97] showed how companies are actually now driving innovation in orthopaedics and MIS was probably one of their greatest successes.

The merit of MIS TKR introduction and its widespread exposure challenged long-term concepts of conventional TKR. This new approach to TKR was driven by several factors. First of all other specialties were ahead of orthopaedic surgery with regard to minimally invasive surgery principles [8]; second of all, the resurgence of UKR using mini-invasive approach gave surgeons the skills to transpose similar technique to TKR [98, 99]; third the development of computer-assisted technology with the navigation and robotic surgery which interestingly followed the progression of UKR growth on the market [12, 97]; and finally the rapid adoption of enhanced recovery thinking in arthroplasty surgery [61, 62].

**Fig. 5:**
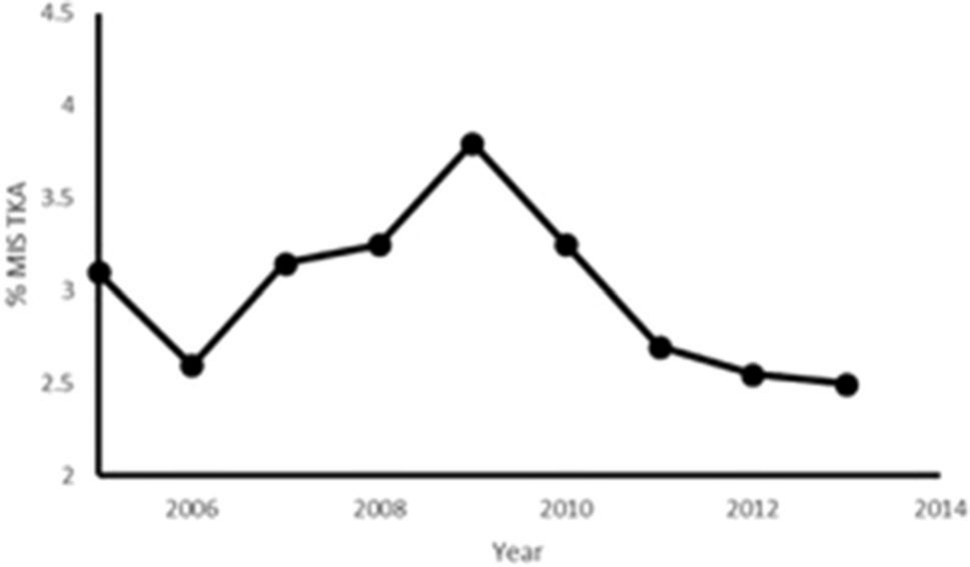
National Joint Registry (NJR) in England reporting MIS Total Knee Arthroplasty (TKA) between 2005 and 2014

Notwithstanding an initial real enthusiasm with MIS TKR, it seems there has been a loss of impetus. The number of MIS TKR in England & Wales recorded in the NJR clearly shows a decrease over recent years [91] (Fig. 5). Despite some authors recommended the use of computer-assisted navigation or PSI to counterbalance the lack of view during MIS TKR and demonstrated good results, both are not mainstream yet [106–109]. One of the main reasons of demoted focus on MIS TKR is the development of the rapid recovery or also called fast track recovery concepts. As given in “MIS approaches description” section, ERPs and LIA used in conventional TKR have proven to give most of the original claimed benefits of MIS. Analysis of the reported improvements of MIS has shown that these are the result of multifactorial changes to surgical practice and there is insufficient evidence that MIS affects early post-operative recovery or soft tissue trauma in isolation to the other included changes [26, 64].

**Fig. 6:**
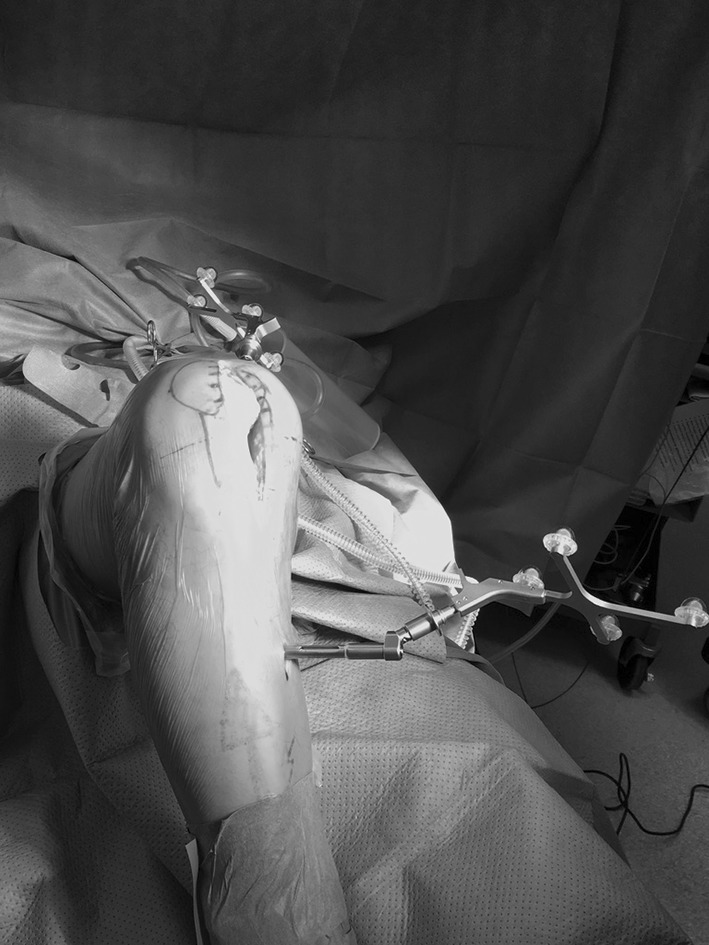
Minimally invasive total knee arthroplasty using computer-assisted navigation technology

Our department, as in many orthopaedic elective high-volume centres, was interested in trying and assessing MIS TKR as a driver to reduce soft tissue invasiveness and subsequently minimize the patient’s recovery time. However, very early on (i.e. in 2007), our department adopted and implanted a very successful enhanced recovery programme [63] including preoperative patient education, local infiltration analgesia (LIA) and early mobilization [100, 101]. We rapidly noticed that even conventional TKR approaches, which were mainly medial parapatellar, allowed the patients to get up and move the same day of surgery and in more than 99% within 24 h with hospital discharge within 4 days [102]. The incentive to continue or implement MIS became less overwhelming for most of the surgeons. Potential disadvantages of less invasive joint replacement related to the difficulty in performing surgery in a restricted visual field and to learning a new-exposure technique had already been widely reported (as given in “MIS approaches description” section) including increased wound problems and risk of malalignment. Our department adopted CAS technology to avoid inaccurate alignment [103], but still MIS TKR lost popularity as the perceived risks outweighed the possible benefits, most of which were already being realized with our ERP (Fig. 6).

It is nevertheless undeniable that MIS steered changes in this field of TKR and the implementation of guided technology and robotic as well as fast recovery programs have benefited from intensively exposed MIS TKR. There is certainly room for MIS or at least LIS for appropriate selected patients. Nonetheless, there are differences between approaches. Mini-medial parapatellar is easy to master, quick to perform and potentially extendable, whereas mini-subvastus and mini-midvastus are maybe trickier and require more caution related to risk of haematoma and VMO nerve damage [104]. Current evidence on the safety and efficacy of mini-incision surgery for TKR does not appear fully adequate for the procedure to be used without special arrangements for consent and for audit or continuing research. There is an argument that a sudden jump from standard TKR to MIS TKR, especially without computer assistance such as navigation, PSI or robotic, may breach a surgeon’s duty of care towards patients because it exposes patients to unnecessary risks [105].

As a final point, more evidence is required on the long-term safety and efficacy of this procedure which will give objective shed light on real benefits of MIS TKR [26, 64].

## Footnotes

1 Aesop. Greek slave and fable author (620 BC–560 BC).

2 Mark Twain. A Connecticut Yankee in King Arthur's Court US humorist, novelist, short story author and wit (1835–1910).

## References

[1] Harrell AG, Heniford BT (2005). Minimally invasive abdominal surgery: lux et veritas past, present, and future. Am J Surg.

[2] Seamon LG, Cohn DE, Henretta MS, Kim KH, Carlson MJ, Phillips GS, Fowler JM (2009). Minimally invasive comprehensive surgical staging for endometrial cancer: robotics or laparoscopy?. Gynecol Oncol.

[3] Daouadi M, Zureikat AH, Zenati MS, Choudry H, Tsung A, Bartlett DL, Zeh HJ (2013). Robot-assisted minimally invasive distal pancreatectomy is superior to the laparoscopic technique. Ann Surg.

[4] Repicci JA, Eberle RW (1999). Minimally invasive surgical technique for unicondylar knee arthroplasty. J South Orthop Assoc.

[5] Judet J, Judet R (1950). The use of an artificial femoral head for arthroplasty of the hip joint. J Bone Joint Surg Br.

[6] Judet J (1952). Technique and results with the acrylic femoral head prosthesis. J Bone Joint Surg Br.

[7] Berger RA (2003). Total hip arthroplasty using the minimally invasive two-incision approach. Clin Orthop Relat Res.

[8] Berry DJ, Berger RA, Callaghan JJ, Dorr LD, Duwelius PJ, Hartzband MA, Mears DC (2003). Minimally invasive total hip arthroplasty. Development, early results, and a critical analysis. J Bone Joint Surg Am.

[9] Tria AJ, Coon TM (2003). Minimal incision total knee arthroplasty: early experience. Clin Orthop Relat Res.

[10] Laskin RS (2003). Session IV: new techniques and concepts in total knee replacement. Clin Orthop Relat Res.

[11] Leitner F, Picard F, Minfelde R, Schulz HJ, Cinquin P, Saragaglia, D (1997) Computer-assisted knee surgical total replacement. In: CVRMed-MRCAS’97. Springer, Berlin, pp 629–638

[12] Delp SL, Stulberg DS, Davies B, Picard F, Leitner F (1998). Computer assisted knee replacement. Clin Orthop Relat Res.

[13] Picard F, Leitner E, Raoult O, Saragaglia D (1999) Computer assisted total knee arthroplasty. In: Rechnergestützte Verfahren in Orthopädie und Unfallchirurgie. Steinkopff, pp 461–471

[14] DiGioia AM, Jaramaz B, Colgan BD (1998). Computer assisted orthopaedic surgery: image guided and robotic assistive technologies. Clin Orthop Relat Res.

[15] Goodfellow JW, Kershaw CJ, Benson MK, O’Connor JJ (1988). The Oxford Knee for unicompartmental osteoarthritis. The first 103 cases. J Bone Joint Surg Br.

[16] DiGioia AM, Plakseychuk AY, Levison TJ, Jaramaz B (2003). Mini-incision technique for total hip arthroplasty with navigation. J Arthroplasty.

[17] Haas SB, Cook S, Beksac B (2004). Minimally invasive total knee replacement through a mini midvastus approach: a comparative study. Clin Orthop Relat Res.

[18] Tenholder M, Clarke HD, Scuderi GR (2005). Minimal-incision total knee arthroplasty: the early clinical experience. Clin Orthop Relat Res.

[19] Fauré BT, Benjamin JB, Lindsey B, Volz RG, Schutte D (1993). Comparison of the subvastus and paramedian surgical approaches in bilateral knee arthroplasty. J Arthroplasty.

[20] Roysam GS, Oakley MJ (2001). Subvastus approach for total knee arthroplasty: a prospective, randomized, and observer-blinded trial. J Arthroplasty.

[21] Laskin RS, Beksac B, Phongjunakorn A, Pittors K, Davis J, Shim JC, Petersen M (2004). Minimally invasive total knee replacement through a mini-midvastus incision: an outcome study. Clin Orthop Relat Res.

[22] Haas SB, Manitta MA, Burdick P (2006). Minimally invasive total knee arthroplasty: the mini midvastus approach. Clin Orthop Relat Res.

[23] Alan RK, Tria AJ (2006). Quadriceps-sparing total knee arthroplasty using the posterior stabilized TKA design. J Knee Surg.

[24] Dalury DF, Dennis DA (2005). Mini-incision total knee arthroplasty can increase risk of component malalignment. Clin Orthop Relat Res.

[25] Jackson G, Waldman BJ, Schaftel EA (2008). Complications following quadriceps-sparing total knee arthroplasty. Orthopedics.

[26] Tria AJ, Scuderi GR (2015). Minimally invasive knee arthroplasty: an overview. World J Orthop.

[27] Gandhi R, Smith H, Lefaivre KA, Davey JR, Mahomed NN (2011). Complications after minimally invasive total knee arthroplasty as compared with traditional incision techniques: a meta-analysis. J Arthroplasty.

[28] Kim YH, Kim JS, Kim DY (2007). Clinical outcome and rate of complications after primary total knee replacement performed with quadriceps-sparing or standard arthrotomy. J Bone Joint Surg Br.

[29] Boonstra RH, Haverkamp D, Campo MM, van der Vis HM (2012). Acute compartment syndrome of the thigh following total knee arthroplasty. Knee.

[30] White RE, Allman JK, Trauger JA, Dales BH (1999). Clinical comparison of the midvastus and medial parapatellar surgical approaches. Clin Orthop Relat Res.

[31] Keating EM, Faris PM, Meding JB, Ritter MA (1999). Comparison of the midvastus muscle-splitting approach with the median parapatellar approach in total knee arthroplasty. J Arthroplasty.

[32] Hofmann AA, Plaster RL, Murdock LE (1991). Subvastus (Southern) approach for primary total knee arthroplasty. Clin Orthop Relat Res.

[33] Keblish PA (1991). Surgical technique and analysis of 53 cases with over two-year follow-up evaluation. Clin Orthop Relat Res.

[34] Repicci JA, Hartman JF (2004). Minimally invasive unicondylar knee arthroplasty for the treatment of unicompartmental osteoarthritis: an outpatient arthritic bypass procedure. Orthop Clin.

[35] Engh GA, Holt BT, Parks NL (1997). A midvastus muscle-splitting approach for total knee arthroplasty. J Arthroplasty.

[36] Tria AJ (2004). Minimally invasive total knee arthroplasty: the importance of instrumentation. Orthop Clin North Am.

[37] Scuderi GR, Tenholder M, Capeci C (2004). Surgical approaches in mini-incision total knee arthroplasty. Clin Orthop Relat Res.

[38] Bonutti PM, Mont MA, McMahon M, Ragland PS, Kester M (2004). Minimally invasive total knee arthroplasty. J Bone Joint Surg.

[39] Rodgers A, Walker N, Schug S, McKee A, Kehlet H, Van Zundert A, Sage D, Futter M, Saville G, Clark T, MacMahon S (2000). Reduction of postoperative mortality and morbidity with epidural or spinal anaesthesia: results from overview of randomised trials. BMJ.

[40] Horlocker TT, Kopp SL, Pagnano MW, Hebl JR (2006). Analgesia for total hip and knee arthroplasty: a multimodal pathway featuring peripheral nerve block. J Am Acad Orthop Surg.

[41] Kerr DR, Kohan L (2008). Local infiltration analgesia: a technique for the control of acute postoperative pain following knee and hip surgery: a case study of 325 patients. Acta Orthop.

[42] Ljungqvist O, Scott M, Fearon KC (2017). Enhanced recovery after surgery: a review. JAMA Surg.

[43] Lonner JH (2006). Minimally invasive approaches to TKA: results. Am J Orthop (Belle Mead NJ).

[44] Insall JN (1984). Surgery of the knee.

[45] Tanavalee A, Thiengwittayaporn S, Itiravivong P (2007). Progressive quadriceps incision during minimally invasive surgery for total knee arthroplasty: the effect on early postoperative ambulation. J Arthroplasty.

[46] Reid JB, Guttmann D, Ayala M, Lubowitz JH (2004). Minimally invasive surgery-total knee arthroplasty. Arthrosc J Arthrosc Relat Surg.

[47] Bonutti PM, Zywiel MG, Ulrich SD, Stroh DA, Seyler TM, Mont MA (2010). A comparison of subvastus and midvastus approaches in minimally invasive total knee arthroplasty. J Bone Joint Surg Am.

[48] Boerger TO, Aglietti P, Mondanelli N, Sensi L (2005). Mini-subvastus versus medial parapatellar approach in total knee arthroplasty. Clin Orthop Relat Res.

[49] Aglietti P, Baldini A, Sensi L (2006). Quadriceps-sparing versus mini-subvastus approach in total knee arthroplasty. Clin Orthop Relat Res.

[50] Seon JK, Song EK (2006). Navigation-assisted less invasive total knee arthroplasty compared with conventional total knee arthroplasty: a randomized prospective trial. J Arthroplasty.

[51] Kolisek FR, Bonutti PM, Hozack WJ, Purtill J, Sharkey PF, Zelicof SB, Rothman RH (2007). Clinical experience using a minimally invasive surgical approach for total knee arthroplasty: early results of a prospective randomized study compared to a standard approach. J Arthroplasty.

[52] Peng X, Zhang X, Cheng T, Cheng M, Wang J (2015). Comparison of the quadriceps-sparing and subvastus approaches versus the standard parapatellar approach in total knee arthroplasty: a meta-analysis of randomized controlled trials. BMC Musculoskelet Disord.

[53] Li C, Zeng Y, Shen B, Kang P, Yang J, Zhou Z, Pei F (2015). A meta-analysis of minimally invasive and conventional medial parapatella approaches for primary total knee arthroplasty. Knee Surg Sports Traumatol Arthrosc.

[54] Xu SZ, Lin XJ, Tong X, Wang XW (2014). Minimally invasive midvastus versus standard parapatellar approach in total knee arthroplasty: a meta-analysis of randomized controlled trials. PLoS ONE.

[55] Liu Z, Yang H (2011). Comparison of the minimally invasive and standard medial parapatellar approaches for total knee arthroplasty: systematic review and meta-analysis. J Int Med Res.

[56] Smith TO, King JJ, Hing CB (2012). A meta-analysis of randomised controlled trials comparing the clinical and radiological outcomes following minimally invasive to conventional exposure for total knee arthroplasty. Knee.

[57] Cheng T, Liu T, Zhang G, Peng X, Zhang X (2010). Does minimally invasive surgery improve short-term recovery in total knee arthroplasty?. Clin Orthop Relat Res.

[58] Alcelik I, Sukeik M, Pollock R, Misra A, Shah P, Armstrong P, Dhebar MI (2012). Comparison of the minimally invasive and standard medial parapatellar approaches for primary total knee arthroplasty. Knee Surg Sports Traumatol Arthrosc.

[59] Kazarian GS, Siow MY, Chen AF, Deirmengian CA (2018). Comparison of quadriceps-sparing and medial parapatellar approaches in total knee arthroplasty: a meta-analysis of randomized controlled trials. J Arthroplasty.

[60] Yuan FZ, Wang SJ, Zhou ZX, Yu JK, Jiang D (2017). Malalignment and malposition of quadriceps-sparing approach in primary total knee arthroplasty: a systematic review and meta-analysis. J Orthop Surg Res.

[61] Kehlet H (1996). Organizing postoperative accelerated recovery programs. Reg Anesth Pain Med.

[62] Jakobsen TL, Kehlet H, Husted H, Petersen J, Bandholm T (2014). Early progressive strength training to enhance recovery after fast-track total knee arthroplasty: a randomized controlled trial. Arthritis Care Res.

[63] McDonald DA, Siegmeth R, Deakin AH, Kinninmonth AWG, Scott NB (2012). An enhanced recovery programme for primary total knee arthroplasty in the United Kingdom—follow up at one year. Knee.

[64] Lloyd JM, Wainwright T, Middleton RG (2012). What is the role of minimally invasive surgery in a fast track hip and knee replacement pathway?. Ann R Coll Surg Engl.

[65] Varela-Egocheaga JR, Suárez-Suárez MA, Fernández-Villán M, González-Sastre V, Varela-Gómez JR, Rodríguez-Merchán C (2010). Minimally invasive subvastus approach: improving the results of total knee arthroplasty: a prospective, randomized trial. Clin Orthop Relat Res.

[66] Bendich I, Moschetti W, Kantor S, Spratt K, Tomek I (2013). Radiographic alignment after minimally invasive total knee arthroplasty: prospective, randomised, controlled trial comparing two approaches. Bone Joint J.

[67] White LD, Melhuish TM (2016). The role of infrapatellar fat pad resection in total knee arthroplasty. Ann Rheum Dis.

[68] Berger RA, Sanders S, Gerlinger T, Della Valle C, Jacobs JJ, Rosenberg AG (2005). Outpatient total knee arthroplasty with a minimally invasive technique. J Arthroplasty.

[69] Chen AF, Alan RK, Redziniak DE, Tria AJ (2006). Quadriceps sparing total knee replacement. J Bone Joint Surg.

[70] McGrory B, Callaghan J, Kraay M, Jacobs J, Robb W, Wasielewski R, Brand RA (2005). Minimally invasive and small-incision joint replacement surgery: what surgeons should consider. Clin Orthop Relat Res.

[71] Hussain SH, Limthongkul B, Humphreys TR (2013). The biomechanical properties of the skin. Dermatol Surg.

[72] Seo JG, Lee SA, Moon YW, Lee BH, Ko YH, Chang MJ (2015). Infrapatellar fat pad preservation reduces wound complications after minimally invasive total knee arthroplasty. Arch Orthop Trauma Surg.

[73] Borges AF (1989). Relaxed skin tension lines. Dermatol Clin.

[74] Piérard GE, Lapière CM (1987). Microanatomy of the dermis in relation to relaxed skin tension lines and Langer’s lines. Am J Dermatopathol.

[75] Weinstein AM, Rome BN, Reichmann WM, Collins JE, Burbine SA, Thornhill TS, Losina E (2013). Estimating the burden of total knee replacement in the United States. J Bone Joint Surg Am.

[76] Niki Y, Mochizuki T, Momohara S, Saito S, Toyama Y, Matsumoto H (2009). Is minimally invasive surgery in total knee arthroplasty really minimally invasive surgery?. J Arthroplasty.

[77] Chang CH, Yang RS, Chen KH, Liu TK, Chen WC, Ho YC, Hou SM (2010). Muscle torque in total knee arthroplasty: comparison of subvastus and midvastus approaches. Knee Surg Sports Traumatol Arthrosc.

[78] Callaghan MJ, Babu VL, Ellis DJ, Samarji RA (2009). Electromyographic comparison of the mid-vastus and sub-vastus approaches to total knee arthroplasty. Curr Orthop Pract.

[79] Kelly MJ, Rumi MN, Kothari M, Parentis MA, Bailey KJ, Parrish WM, Pellegrini VD (2006). Comparison of the vastus-splitting and median parapatellar approaches for primary total knee arthroplasty: a prospective, randomized study. J Bone Joint Surg.

[80] Ghosh KM, Hunt N, Blain A, Athwal KK, Longstaff L, Amis AA, Deehan DJ (2015). Isolated popliteus tendon injury does not lead to abnormal laxity in posterior-stabilised total knee arthroplasty. Knee Surg Sports Traumatol Arthrosc.

[81] Kesman TJ, Kaufman KR, Trousdale RT (2011). Popliteus tendon resection during total knee arthroplasty: an observational report. Clin Orthop Relat Res.

[82] Reid MJ, Booth G, Khan RJ, Janes G (2014). Patellar eversion during total knee replacement: a prospective, randomized trial. J Bone Joint Surg.

[83] Kuo FC, Lin PC, Lu YD, Lee MS, Wang JW (2017). Chronic kidney disease is an independent risk factor for transfusion, cardiovascular complication, and thirty-day readmission in minimally invasive total knee arthroplasty. J Arthroplasty.

[84] Ang CL, Yeo SJ (2016). Quality of cementation in conventional versus minimally invasive total knee arthroplasty. J Orthop Surg (Hong Kong).

[85] Lüring C, Beckmann J, Haiböck P, Perlick L, Grifka J, Tingart M (2008). Minimal invasive and computer assisted total knee replacement compared with the conventional technique: a prospective, randomised trial. Knee Surg Sports Traumatol Arthrosc.

[86] Biasca N, Wirth S, Bungartz M (2009). Mechanical accuracy of navigated minimally invasive total knee arthroplasty (MIS TKA). Knee.

[87] Seon JK, Song EK, Yoon TR, Park SJ, Bae BH, Cho SG (2007). Comparison of functional results with navigation-assisted minimally invasive and conventional techniques in bilateral total knee arthroplasty. Comput Aided Surg.

[88] Ng VY, DeClaire JH, Berend KR, Gulick BC, Lombardi AV (2012). Improved accuracy of alignment with patient-specific positioning guides compared with manual instrumentation in TKA. Clin Orthop Relat Res.

[89] Aglietti P, Baldini A, Giron F, Sensi L (2009). Minimally invasive total knee arthroplasty: is it for everybody?. HSS J.

[90] Watanabe T, Muneta T, Ishizuki M (2009). Is a minimally invasive approach superior to a conventional approach for total knee arthroplasty? Early outcome and 2-to 4-year follow-up. J Orthop Sci.

[91] Unwin O, Hassaballa M, Murray J, Harries W, Porteous A (2017). Minimally invasive surgery (MIS) for total knee replacement; medium term results with minimum five year follow-up. Knee.

[92] Khakha RS, Chowdhry M, Norris M, Kheiran A, Patel N, Chauhan SK (2014). Five-year follow-up of minimally invasive computer assisted total knee arthroplasty (MICATKA) versus conventional computer assisted total knee arthroplasty (CATKA)—a population matched study. Knee.

[93] Klingenstein G, Porat M, Elsharkawy K, Reid J (2017). Evaluating the impact of morbid obesity on minimally invasive total knee replacement patients. Bone Joint J.

[94] Amanatullah DF, Burrus MT, Sathappan SS, Levine B, Di Cesare PE (2012). The application of minimally invasive surgical techniques. Part II: total knee arthroplasty. Am J Orthop (Belle Mead NJ).

[95] Wickham JEA (1994). Minimally invasive surgery: future developments. BMJ.

[96] Callaghan JJ, Warth LC, Liu SS, Hozack WJ, Klein GR (2006). Internet promotion of MIS and CAOS in TKA By Knee Society members. Clin Orthop Relat Res.

[97] Dalton DM, Burke TP, Kelly EG, Curtin PD (2016). Quantitative analysis of technological innovation in knee arthroplasty: using patent and publication metrics to identify developments and trends. J Arthroplasty.

[98] Tria AJ (2002). Minimally invasive unicompartmental knee arthroplasty. Tech Knee Surg.

[99] Pandit H, Jenkins C, Barker K, Dodd CAF, Murray DW (2006). The Oxford medial unicompartmental knee replacement using a minimally-invasive approach. J Bone Joint Surg Br.

[100] Quinn M, Deakin AH, McDonald DA, Cunningham IKT, Payne AP, Picard F (2013). An anatomic study of local infiltration analgesia in total knee arthroplasty. Knee.

[101] Brydone AS, Souvatzoglou R, Abbas M, Watson DG, McDonald DA, Gill AM (2015). Ropivacaine plasma levels following high-dose local infiltration analgesia for total knee arthroplasty. Anaesthesia.

[102] McDonald DA, Deakin AH, Ellis BM, Robb Y, Howe TE, Kinninmonth AWG, Scott NB (2016). The technique of delivery of peri-operative analgesia does not affect the rehabilitation or outcomes following total knee arthroplasty. Bone Joint J.

[103] Smith BR, Deakin AH, Baines J, Picard F (2010). Computer navigated total knee arthroplasty: the learning curve. Comput Aided Surg.

[104] Holt G, Nunn T, Allen RA, Forrester AW, Gregori A (2008). Variation of the vastus medialis obliquus insertion and its relevance to minimally invasive total knee arthroplasty. J Arthroplasty.

[105] Holt G, Wheelan K, Gregori A (2006). The ethical implications of recent innovations in knee arthroplasty. J Bone Joint Surg.

[106] Dutton AQ, Yeo SJ, Yang KY, Lo NN, Chia KU, Chong HC (2008). Computer-assisted minimally invasive total knee arthroplasty compared with standard total knee arthroplasty: a prospective, randomized study. J Bone Joint Surg.

[107] Hafez MA, Chelule KL, Seedhom BB, Sherman KP (2006). Computer-assisted total knee arthroplasty using patient-specific templating. Clin Orthop Relat Res.

[108] Zhu M, Ang CL, Chong HC, Yeo SJ (2016). Computer-assisted minimally invasive total knee arthroplasty compared with conventional total knee arthroplasty: a prospective nine-year follow-up. Bone Joint J.

[109] Hasegawa M, Miyazaki S, Yamaguchi T, Wakabayashi H, Sudo A (2017). Comparison of midterm outcomes of minimally invasive computer-assisted vs minimally invasive jig-based total knee arthroplasty. J Arthroplasty.

